# Optimizing osmotic pressure removes EBV particles from B95-8 host cells while maintaining normal activity

**DOI:** 10.3892/etm.2013.907

**Published:** 2013-01-17

**Authors:** MIN PAN, JING SHEN, JIE CAI

**Affiliations:** 1 Department of Clinical Laboratory, First Affiliated Hospital of Guangxi Medical University; Nanning, Guangxi 530021, P.R. China; 2 Experimental Center of Medical Sciences, Guangxi Medical University, Nanning, Guangxi 530021, P.R. China

**Keywords:** Epstein-Barr virus, B95-8 cell, optimal osmotic pressure

## Abstract

This study demonstrates the removal of virus particles from B95-8 host cells that maintain normal activity under optimal osmotic pressure. After infecting B95-8 cells with Epstein-Barr virus (EBV) particles, the cells were treated with isosmotic solution [0.90% NaCl (330 mOsm/kg H_2_O)], hyposmotic solutions [0.36% NaCl (115 mOsm/kg H_2_O) and 0.27% NaCl (93 mOsm/kg H_2_O)] and distilled water. The pumping levels of virus particles were observed by inverse phase contrast microscopy and transmission electron microscopy (TEM). After treatment with the hyposmotic solutions, the following results were observed: firstly, after culturing for 24 and 48 h, the B95-8 cells in the hyposmotic solutions grew as well as the cells cultured in the isosmotic solution. Secondly, the virus particles in the B95-8 host cells overflowed onto the surface of the cells, while the organelle structures remained intact. This phenomenon was repeated in the removal of human immunodeficiency virus (HIV) from leukomonocytes. By optimizing the osmotic pressure, the activity of the B95-8 host cells was retained and the EBV particles were transported from the cells onto the cell surface.

## Introduction

The cell membrane is a semipermeable membrane. Low levels of penetrating fluid are likely to dissolve cells, particularly red blood cells, and the excessive dissolution of red blood cells results in kidney damage. In the process of metabolism and a variety of physiological functions, a flow of active molecules in and out of the cell occurs and the cell volume changes. Excessive volume change leads to cellular dysfunction (1). However, cells are partly able to influence the changes in cell volume. The tolerance of cells to low osmotic pressures and semipermeable membrane permeability is different (2). To date, with the exception of blood cells, changes to cells under varying osmotic pressures have not been reported. In this study, B95-8 cell growth, membrane changes and Epstein-Barr virus (EBV) infection were investigated under varying osmotic pressures [0.90% NaCl (330 mOsm/kg H_2_O), 0.36% NaCl (115 mOsm/kg H_2_O), 0.27% NaCl (93 mOsm/kg H_2_O) and distilled water]. In addition, under the above pressure conditions, we investigated whether the virus was pumped out onto the cell surface, to allow the virus to be effectively cleared by drugs. Additionally, we investigated the removal of the human immunodeficiency virus (HIV) from lymphocytes at a low osmotic pressure.

## Materials and methods

### Materials

#### Electronic instruments

The following equipment was used: microscope (Hitachi, Tokyo, Japan), H-500Steri-cycle (Hitachi), Sanyo MC0175 CO_2_ incubator (Sanyo, Osaka Moriguchi, Japan), SW-CJ-IF super-clean single-sided platform (Aertai, Suzhou, China), inverted biological microscope (Chongqing Optical and Electrical Instrument Co., Ltd., Chongqing, China) and an advanced model 3900 osmometer (Advanced Instruments, Norwood, MA, USA).

#### Solutions

The following solutions were prepared in a sterile environment: 0.90% NaCl solution (330 mOsm/kg H_2_O, isotonic solution), 0.36% NaCl solution (115 mOsm/kg H_2_O, hypotonic solution), 0.27% NaCl solution (93 mOsm/kg H_2_O, hypotonic solution) and distilled water. In addition, excess solution was used to measure the osmotic pressure using the osmometer.

#### Stain

A solution of trypan blue (0.4%) was prepared with normal saline.

#### B95-8 cells

B95-8 cells were obtained from the Chinese Medical Science Institute. For cell recovery, the following procedure was performed: the cryopreservation tubes were removed from the liquid nitrogen tank and quickly placed in a 37°C water bath, with shaking from time to time. The freezing tube was scrubbed with 75% ethanol. The cell suspensions were then removed and injected into a centrifuge tube, dropping the culture solution 10 times to mix. The solution was centrifuged at low-speed (1,000 rpm) for 10 min, then the supernatant was discarded and cells were washed repeatedly with RPMI-1640 medium to remove the dimethyl sulfoxide. The culture solution was transferred to a culture bottle and placed into the CO_2_ incubator (37°C, 5% CO_2_). The following day, the culture medium was replaced. The cells were collected after culturing for 7 days. The number of cells was determined to be 1×10^7^/ml by electron microscopy (3).

### Methods

#### B95-8 cells under various osmotic pressures

Under sterile conditions, B95-8 cells were placed into 10 ml centrifuge tubes containing 0.90% NaCl solution (330 mOsm/kg H_2_O, isotonic solution), 0.36% NaCl solution (115 mOsm/kg H_2_O), 0.27% NaCl solution (93 mOsm/kg H_2_O, hypotonic solution) or distilled water, respectively. The cells were cultured at 37°C for 5 min and then centrifuged at low-speed (1,000 rpm) for 5 min. The supernatant was discarded and the step was repeated twice. The cells were then centrifuged at 2,000 rpm for 5 min and the B95-8 cells collected. The cells from each solution were then placed into three tubes. The total solution in each tube was 0.3 ml and the density of the cells was 5×10^6^/ml. To each tube, 20 *μ*l 0.4% trypan blue was added for 10 min. The blood cells were counted on a counting plate under a common microscope. The blue-stained cells were dead and the cells without staining were living. In each tube, 500 cells were counted and the percentage of surviving cells was calculated. The cells were placed in a 24-well plate to culture for 48 h in RPMI-1640 medium. The cells were fixed with 3% glutaraldehyde fixation overnight then washed with buffer, acidized with l% osmium tetroxide, dehydrated with an ethanol gradient, embedded with epoxy resin 618, cut into sections using an LKB-V slicer and stained using uranyl acetate and lead citrate (4).

#### HIV cells and low osmotic pressure

This study was approved by the ethics committee of Guangxi medical university, Nanning, China. After obtaining informed consent from patients, 10 ml blood was extracted from untreated HIV-positive patients. The blood was divided into three tubes. Each tube contained 3 ml venous blood and was mixed with 3 ml Hanks’ fluid to dilute. Then, 3 ml diluted blood was added to 2 ml lymphocyte separation liquid and centrifuged at 2,000 rpm for 20 min. A flat capillary tube was used to absorb the single nuclear cells, which were transferred into a short tube. More than five times the volume of Hanks’ solution was added and the cells were washed twice. The concentrated nucleated cells were placed in a tube. Some of these cells were placed in a 24-well plate and cultured for 48 h in RPMI-1640 medium. The remainder were fixed with 3% glutaraldehyde and laid aside overnight to prepare for electronic microscopic examination. The cells were fixed with l% osmium tetraoxide, dehydrated using an alcohol gradient, embedded in epoxy resin 618, cut into sections using an LKB-V slicing machine and double stained with uranyl acetate and lead citrate.

## Results

### 

#### Trypan blue staining

The survival rates of 500 cells under varying osmotic pressures as revealed by trypan blue staining are shown in Table I.

No difference in the cell survival rate among the different osmotic pressures was observed. Cells swelled with low permeability in the low osmotic pressure tube. The blue cells were considered to be apoptotic cells arising from the process of cultivation. In addition, there were a small number of surviving cells in the distilled water treatment tube.

#### State of the cells under an inverted microscope at different osmotic pressures

**Figure 1.**
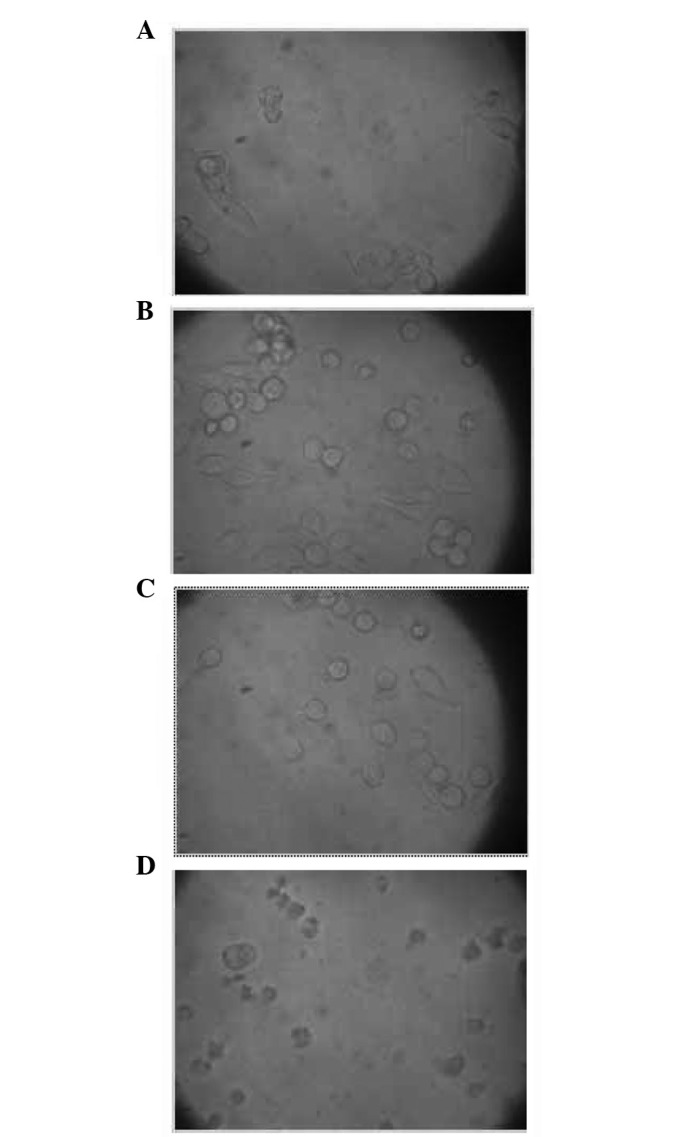
Growth of the B95-8 cells under varying osmotic pressures. (A) 0.90% NaCl solution (330 mOsm/kg H_2_O). (B) 0.36% NaCl solution (115 mOsm/kg H_2_O). (C) 27% NaCl solution (93 mOsm/kg H_2_O). (D) Distilled water. Magnification, 10×40.

Cells were observed under the inverted microscope with four different osmotic pressures, 0.90% NaCl solution (330 mOsm/kg H_2_O, isotonic solution), 0.36% NaCl solution (115 mOsm/kg H_2_O, hypotonic solution), 0.27% NaCl solution (93 mOsm/kg H_2_O, hypotonic solution) and distilled water. After experiencing the altered osmotic pressure for 48 h, B95-8 cells maintained normal activity. However, the majority of cells treated with distilled water stopped growing (Fig. 1).

#### Cell states under different osmotic pressures under a transmission electron microscope

**Figure 2.**
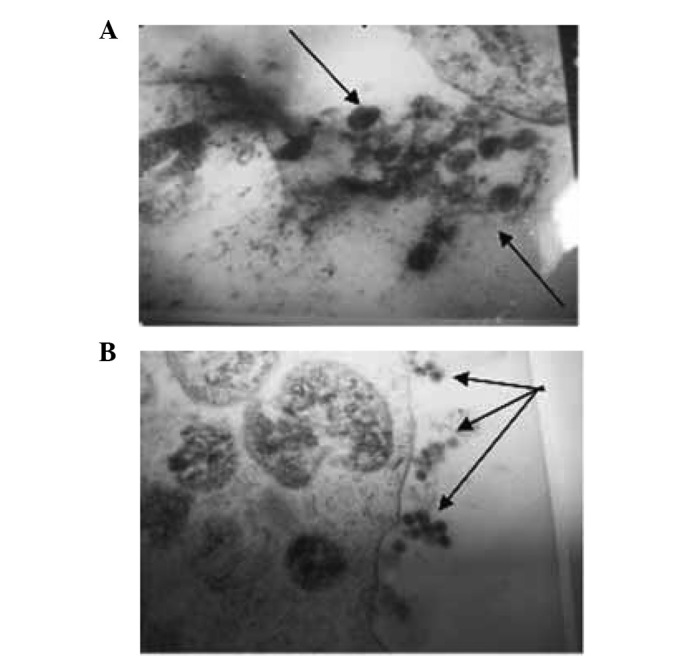
Transmission electron microscopy results following treatment in 0.90% NaCl solution (330 mOsm/kg H_2_O). (A) Virus particles (arrows) were observed in the cytoplasm (magnification, ×30,000). (B) Virus particles (arrows) were observed on the cell surface (magnification, ×12,000).

**Figure 3.**
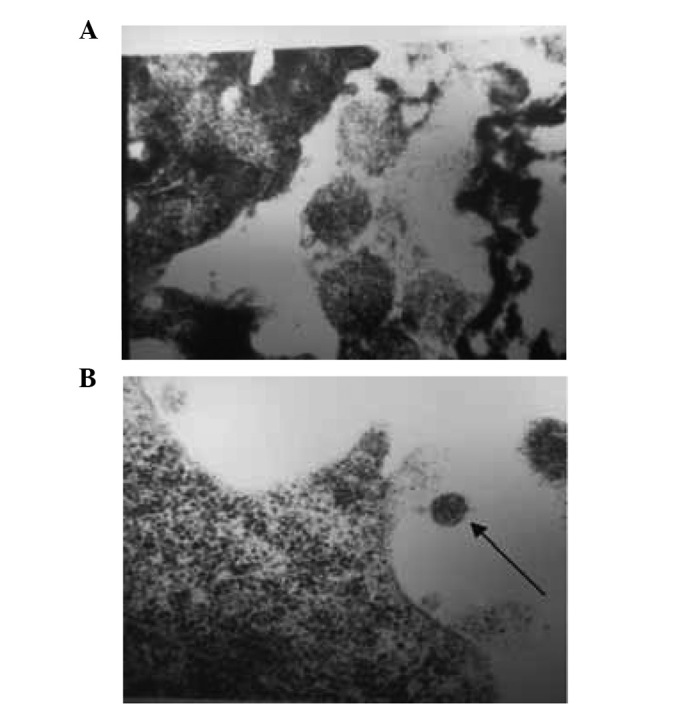
Transmission electron microscopy results following treatment in 0.36% NaCl solution (115 mOsm/kg H_2_O). (A) Normal rough endoplasmic reticulum was observed around the cytoplasm (magnification, 20,000). (B) Virus particles (arrow) were observed on the cell surface (magnification, ×32,000).

**Figure 4.**
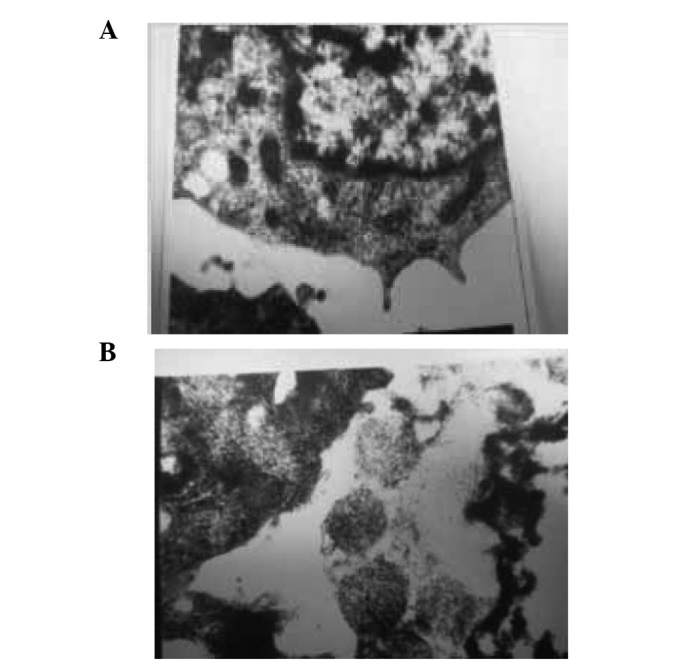
Transmission electron microscopy results following treatment in 0.27% NaCl solution (93 mOsm/kg H_2_O). (A) Normal endoplasmic reticulum, mitochondria and chorion were observed (magnification, ×10,000). (B) Normal endoplasmic reticulum and mitochondria were observed (magnification, ×10,000).

**Figure 5.**
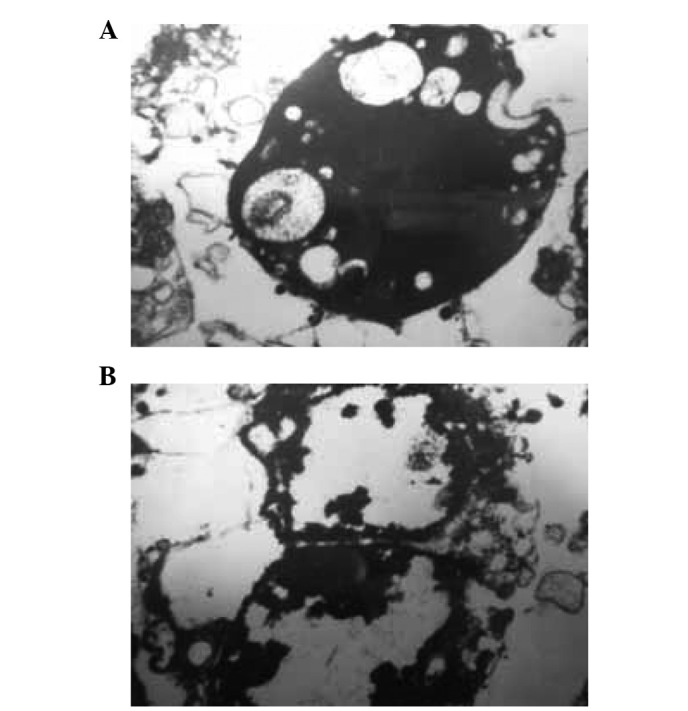
Transmission electron microscopy results following treatment in distilled water. (A) Nuclear chromatin gathering and cell microvilli disappeared (magnification, ×4,800). (B) A pyknotic nucleus was observed and the cytoplasm contained large vacuoles (magnification, ×4,800).

Following treatment in the isotonic solution [0.90% NaCl solution (330 mOsm/kg H_2_O)], EBV particles were observed to be located inside and outside the cells. The whole cell structure is shown in Fig. 2. Following treatment in 0.36% NaCl solution (115 mOsm/kg H_2_O), the organelle structure of the B95-8 cells remained intact (Fig. 3). In the 0.27% NaCl solution (93 mOsm/kg H_2_O), EBV particles were not observed in the B95-8 cells (Fig. 4). In addition, the intracellular organelles of the B95-8 cells were observed to have become vacuolated following treatment with distilled water (Fig. 5).

#### Pre-experiment of HIV virus particle pumping from human lymphocytes under low osmotic pressure

**Figure 6.**
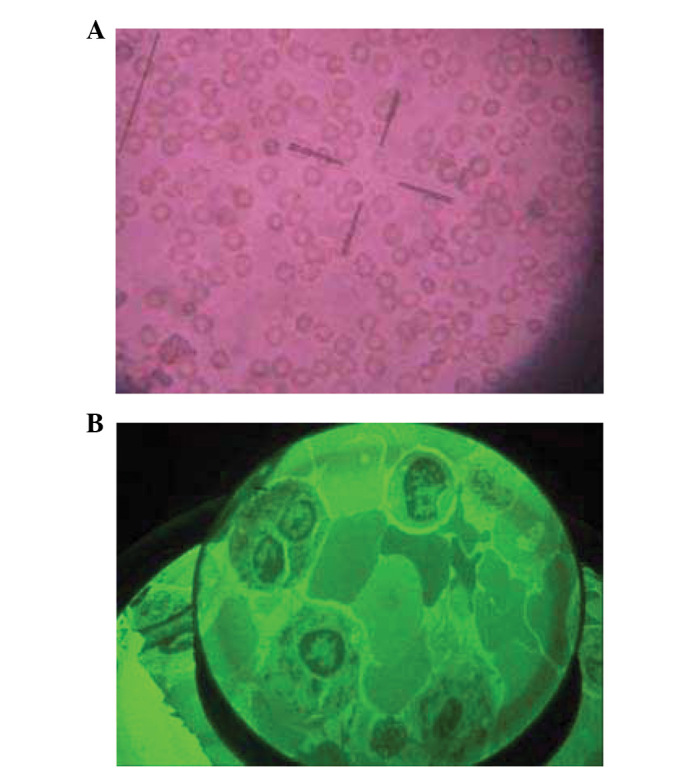
Blood cells in a hypotonic solution [0.30% NaCl (104 mOsm/kg H_2_O)]. (A) Normal cell growth and activity (magnification, 10×40). (B) Lymphoid/myeloid (magnification, ×10,000).

We investigated whether HIV is removed from lymphocytes under low osmotic pressure. We observed that in 0.30% NaCl solution (104 mOsm/kg H_2_O), HIV virus particles were not present in the CD4^+^ T cells. In addition, as was observed for the removal of EBV from B95-8 cells, following treatment in a hypotonic solution [0.30% NaCl (104 mOsm/kg H_2_O)] for 48 h, some of the cells maintained normal growth and activity (Fig. 6A). Observation of cells, under an electron microscope, revealed that the main organelles retained structural integrity and did not present any changes (Fig. 6B). In the two images, we observed that a number of red blood cells did not dissolve following treatment in the hypotonic solution [0.30% NaCl (104 mOsm/kg H_2_O)].

In the subsequent experiments using the CD^+^4 cells, we observed that a number of the red blood cells in the moderately hypotonic solution [0.45% NaCl (140 mOsm/kg H_2_O)] began to burst and underwent hemolysis. The membranes of the red blood cells in the more hypotonic solution [0.27% NaCl (93 mOsm/kg H_2_O)] were almost completely broken and dissolved.

## Discussion

Osmosis is a common phenomenon in nature and is important for maintaining the normal physiological function of the human body. The semipermeable membrane is a selectively permeable membrane and excessive penetration of liquid leads to cell lysis (5). The current study aimed to maintain the B95-8 host cell activity while pumping out the EBV under variable osmotic pressure. Our results demonstrated that the cells treated with a suitable osmotic pressure maintained normal activity and the main structures in the cell’s complement system were not altered. The cell underwent normal growth. In addition, virus particles were pumped to the cell surface. B95-8 cells are commonly used as the target cell for EBV. Under equal osmotic pressure and low osmotic pressure, viral growth and maturation did not lead to apoptosis and cell rupture. The virus was no longer in the cell. In addition, at a low osmotic pressure, a number of virus particles may have been collected by centrifugation or have been pumped out of the cell. These results provide an important theoretical basis for the effective removal of intracellular viruses from cells.

Different cells have different hypotonic tolerances and membrane permeability. Red blood cells are more sensitive at low osmotic pressures. We conclude that, when treated with 0.30% NaCl solution (104 mOsm/kg H_2_O), some of the cells maintain normal activity following 48 h culture and under an electron microscope, the structure of cell organelles maintain integrity. However, a number of red blood cells were swollen. In addition, the red blood cells began to rupture and dissolve in the moderately hypotonic solution [0.45% NaCl (140 mOsm/kg H_2_O)]. In the more hypotonic solution [0.27% NaCl (93 mOsm/kg H_2_O)], the B95-8 cells were almost completely ruptured and dissolved. Therefore, in order to pump HIV out of the cell, the exploration of a suitable low osmotic concentration is required. Additionally, further research is required to determine whether the virus that has been pumped out is capable of infecting again.

Studies of osmotic pressure have only investigated red blood cells. Osmotic pressure is divided into three levels: absolute hypertonic, hypotonic and isotonic. According to our experimental results, it is more reasonable that the cell osmotic pressure be divided into 5 levels and that there are buffer zones from the low osmotic pressure to the osmotic pressure and from the osmotic pressure to the high osmotic pressure. Therefore, the levels would be: i) high osmotic pressure: all intracellular fluid passively penetrates outwards, so cell structure and function are destroyed; ii) neutral-high osmotic pressure: a small amount of intracellular fluid passively penetrates outwards and the cell wall has a certain degree of shrinkage although the cell structure and function are complete and the cell grows well after culturing; iii) equal osmotic pressure: cell function and morphology do not change, the exchange of substance is decided by the needs of the cell and the exchange speed of intracellular and extracellular fluid is slow; iv) neutral-low osmotic pressure: hypotonic solution flows into the cell, cell swelling is observed, the cell structure and function are complete, the cell grows well after culturing, and some cell fluid, parasitic virus particles and the protein in the cytosol produced before and after maturation of the virus are pumped out and v) low osmotic pressure: cell structure and function are damaged, most of the membrane is dissolved, organelles enter the vacuole and cell death occurs.

A relatively stable internal environment is provided by the cell membrane. The semipermeable membrane selectively transports metabolites into and out of the cell, according to the needs of the cell. There are four forms of liquid exchange: i) liquid is blocked and cannot be exchanged; ii) cells are selectively exchanged via the semipermeable membrane according to the needs of the cell, but the speed is slow; iii) low-molecular-weight molecules are exchanged quickly as in hypotonic treatment and iv) barrier-free exchange. A relatively large number of substances are quickly and controllably transported to the intracellular environment. Therefore, it is possible to block viral synthesis in the chromosomes. HIV-1 patients may be able to receive early intervention therapy prior to symptom formation. This study demonstrates the effect of the low osmotic pressure theory.

Host cell function is not affected and the virus particles are successfully removed from the cells. We consider that relative to the host cell, as a foreign protein, the virus particles do not have not a solid attachment to the host cell; therefore, low osmotic pressure may be effectively used to pump out the virus into the extracellular space.

## Figures and Tables

**Table I.** Survival rates of cells under different osmotic pressures.

Osmotic pressure	Cell survival rate (%)
0.90% NaCl (330 mOsm/kg H_2_O)	92
0.36% NaCl (115 mOsm/kg H_2_O)	89
0.27% NaCl (93 mOsm/kg H_2_O)	91
Distilled water	2
